# Video-endoscopic Tension-free Groin Hernia Repair via Total Extraperitoneal Approach

**DOI:** 10.7759/cureus.6839

**Published:** 2020-02-01

**Authors:** Sami Dogan, Emin Gurleyik

**Affiliations:** 1 Surgery, Duzce University Medical Faculty, Düzce, TUR

**Keywords:** inguinal, tep approach, recurrence, prolene mesh

## Abstract

Introduction

Tension-free repair of groin hernias with synthetic material by video-endoscopic surgery is a widely accepted method that is performed by various approaches. We aim to present our results of video-endoscopic tension-free repair of groin hernias via the total extraperitoneal (TEP) approach.

Methods

Between September 2016 and December 2018, 124 patients with groin hernias underwent tension-free repair using prolene mesh by video-endoscopic surgery via the TEP approach. This is a retrospective analysis of 110 (88.7%) male and 14 (11.3%) female patients with a mean age of 48.1 years. Groin hernia types, locations of hernias, intraoperative and postoperative complications, results of the mean 24-month follow-up, and recurrence rate were reported.

Results

A total of 134 hernias were repaired in 124 patients who had 53 (42.7%) right, 61 (49.2%) left, and 10 (8.1%) bilateral groin hernias. The most common hernia type was an indirect inguinal hernia in 83/134 (62%) groin hernias. A total of 119 (88.8%) and 15 (11.2%) hernias were primary and recurrent, respectively. Seroma was detected in two (1.6%) patients in the early postoperative period. The mean duration of hospital stay was 1.4 (1-3) days. During the follow-up period, hernia recurrence was determined in three (2.4%) patients. Hernia recurrence was detected among patients who were operated on during the first half of the study.

Conclusion

Tension-free repair of groin hernias by video-endoscopic technique via the TEP approach can be performed with very low complication and recurrence rates. The success of the TEP approach increases parallel to increasing surgical experience. The results of hernia repair via the TEP approach are highly satisfactory and encouraging, especially when attention is paid to proper patient selection during the learning curve period.

## Introduction

Tension-free repairs are the current methods of choice in groin hernia surgery because they have a more comfortable postoperative course and lower recurrence rates. Tension-free repairs using synthetic material via video-endoscopic interventions are widely accepted in hernia surgery [1]. Transabdominal preperitoneal (TAPP) and total extraperitoneal (TEP) methods with a posterior approach have been implemented as video-endoscopic techniques. The video-endoscopic TEP technique seems to be preferable because it confers the advantage of protecting intra-abdominal tissues. The surgical objective of all open and video-endoscopic tension-free hernia repairs with the posterior approach is to strengthen the posterior wall of the inguinal or femoral canal using synthetic mesh. The TEP approach is an excellent method for this purpose [2,3]. The first 50 patients were treated with the extraperitoneal placement of prosthetic material via a laparoscope from 1989 to 1991. Today, this technique, known as TEP, has become widespread as noninvasive endoscopic surgery [4]. Video-laparoscopic hernia surgery has the following advantages: less pain, faster recovery, good cosmetic results, and an earlier return to normal life and work. In addition, since there is no peritoneal opening, the TEP technique is seen as a minimally invasive method with the lowest risk of injury to intra-abdominal organs.

The aim of this study was to investigate the efficacy of our video-endoscopic TEP technique in the surgical treatment of groin hernias.

## Materials and methods

Between September 2016 and December 2018, a total of 128 patients were scheduled for groin hernia surgery by the TEP approach. Four patients were excluded because of conversion to other techniques (three to the TAPP technique and one to open surgery). Thus, a total of 124 patients with groin hernia were surgically treated with the video-laparoscopic TEP technique.

Surgical technique 

Video-endoscopic TEP repair was performed under general anesthesia using the three-port technique. The preperitoneal area was expanded using 10-mm balloon dilatators (AutoSuture Spacemaker Plus®, Covidien™, Mansfield, MA) inserted through a 1.5-cm incision made from the midline, passing through the skin, subcutaneous and rectus anterior sheath, and then through the rectus sheaths to the symphysis pubis. Within the preperitoneal space, the Bogros space was expanded by inflating the balloon. The patient was then placed in the Trendelenburg position at approximately 30° laterally. In each case, the pubis, Cooper’s ligament, and rectus muscle fibers were visualized and identified through the balloon, and then the balloon was deflated and removed. After the CO_2_ insufflation was set to a maximum pressure of 12 mmHg through the bottom hub port, two additional 5 mm trocars were placed in the midline about 5 cm below the margin of the umbilicus, under the 30° camera view angle. The dissection area was enlarged until the Cooper’s ligament, symphysis pubis, and inferior epigastric vessels were seen on the inner side, and the outer edge of the psoas muscle and the iliopubic line appeared at the anterior superior level on the outer side of the iliac spine. Reduction of the entire hernia sac and ligation of its peritoneal opening was performed after the rotation of the hernia sac at the level of the inner inguinal ring, and separation of the spermatic cord and vessels was achieved in male cases. A prolene patch that had been prepared in the appropriate dimensions (15 cm x 15 cm) was positioned to cover the potential hernia areas by passing the mesh through an incision from medial to lateral under the cord structures and ensuring to cover the inner ring from the lateral borders to the top. The hemorrhage and pain triangles were preserved, and it was anchored with absorbable mesh tacks to the inner side of the pubic bone and Cooper’s ligament, on either side of the upper-middle and inferior epigastric vessels, and to the outer side of the transverse aponeurosis at the level of the iliac bone. Under the view of the camera, the process was terminated by providing gas desufflation.

The hemorrhage triangle (triangle of iliac vessels): Medial and lateral borders of this triangle are vas deferens and spermatic vessels respectively. This triangle corresponds to the location of external iliac vessels.

The pain triangle (triangle of the nerves): Medial border is formed by spermatic vessels, superior and lateral borders are formed by the iliopubic tract. This triangle is known as passage of the nerves; lateral cutaneous nerve of the thigh, femoral branch of genitofemoral nerve and femoral nerve. 

The demographic data, hernia type, intra- and postoperative complications, postoperative pain, normal life, and return to work status of these cases were evaluated retrospectively. The mean follow-up period was 24 months (range, 12-36 months). Patients underwent clinical follow-ups at one, three, and six months and annually. All patients were examined for recurrence. Surgical success was evaluated by physical examination and ultrasound imaging in suspicious cases.

## Results

Of the 124 patients with groin hernias who underwent the TEP technique, 110 (88.7%) were male with a mean age of 48.1 years. Sixty-one (49.2%) patients had a left inguinal hernia alone. Of 134 groin hernia sides, including 10 bilateral cases, 71 left, and 63 right groin hernias were repaired with the TEP approach. Fifteen (12.1%) patients underwent tension-free repair with the TEP technique for recurrent hernia (Table 1).

**Table 1:** Location of hernia in patients with groin hernia

Location	Patients (n=124)	Groin hernia (n=134)
Right	53 (42.7%)	63 (47%)
Left	61 (49.2%)	71 (53%)
Bilateral	10 (8.1%)	
Total	124 (100%)	134 (100%)

Eighty (64.5%) patients had indirect inguinal hernias. Eighty three (62%) of 134 groin hernias were indirect inguinal hernias (Table 2).

**Table 2:** Groin hernia types

Groin hernia types	Patients (%)	Groin hernia (%)
Direct inguinal	37 (29.9)	44 (32.8)
Indirect inguinal	80 (64.5)	83 (62)
Femoral	3 (2.4)	3 (2.2)
Combined	4 (3.2)	4 (3.0)
Total	124 (100)	134 (100)
Primary hernia	109 (87.9)	119 (88.8)
Recurrent hernia	15 (12.1)	15 (11.2)

None of our patients experienced pain requiring medication postoperatively. Seroma was detected in 2 (1.6%) patients as a surgical complication in the early postoperative period. The mean hospital stay was 1.4 days (range, 1-3 days). The mean time of return to work was seven days (range, 4-12 days). Recurrence of hernias has occurred in three (2.4%) patients with indirect inguinal hernias in the follow-up period. Our patients with hernia recurrence were operated on during first half of the study.

## Discussion

Before the development of video-endoscopic techniques, the tension-free repair was reported to be the most successful method in open hernia surgery. Previously, hernia repair was performed with minimum tension using the tissues of the body, while supplementary use of synthetic material provided complete tensionless repair. In recent years, repairs using video-endoscopic methods and synthetic materials have become increasingly successful and widely accepted. Today, the most widely used video-endoscopic methods are TEP and TAPP. The advantage of TEP is that the peritoneal cavity is not opened. Thus, intraperitoneal complications are minimized. However, the narrowness of the working area can sometimes be a disadvantage. In this study, the findings of our patients who underwent groin hernia repair via the TEP approach were investigated. The most important advantage of the TEP technique in this series of patients was the avoidance of intraperitoneal complications due to an intact peritoneal cavity.

According to our preoperative findings, a technical conversion was required in 4/128 (3.1%) cases. In our series, one (0.8%) patient with a recurrent hernia, who had previously undergone video-endoscopic intervention, required conversion to open surgery. Except for this patient who was converted to open surgery, the surgery was completed with the video-endoscopic TAPP technique in three (2.3%) patients. In one large series with long-term results, the conversion rate of the video-endoscopic TEP technique was reported in 13/412 (3.2%) patients [5]. An important parameter in the early postoperative period is the presence of acute and chronic pain. Our patients did not require analgesic medication postoperatively. Less chronic pain has been reported with the TEP technique than with other methods [6,7]. Seroma formation was the most common early surgical site complication in our series.

Similarly, in the literature, seroma (3.4%) and hematoma (1.2%) have been reported as early complications of the TEP approach [8,9]. The short length of the hospital stay in our series of groin hernias with the TEP repair confirms the advantage of video-endoscopic tension-free methods. In a study comparing TEP with open methods, the lengths of hospital stay were 1.6 and 3.1 days for the TEP approach and the open prolene mesh hernioplasty, respectively. The important advantages of endoscopic repairs compared with the open methods are short recovery time, early return to work, and good cosmetic results. Another reported advantage is the finding and repair of unexpected hernias by video-endoscopic exploration, thus reducing possible recurrence rates [10-12].

Early and late recurrence rates are probably the most important findings for the effectiveness of hernia repair techniques. In our study, lower recurrence rates during follow-up of two years support the success of the video-endoscopic tension-free repair. In previous studies, the recurrence rate was found to be between 0.1% and 4% [13,14]. In tension-free techniques, the posterior wall repair was performed with a synthetic mesh material that may tend to migrate or cause a hernia to recur. However, according to the results of two previous randomized studies, no relationship between mesh fixation and the recurrence rate was found.

In contrast, hernia repair with free mesh without fixation has been reported to reduce postoperative complications and to be cost-effective compared with fixation techniques [15]. Recently, recurrence rates of less than 0.3% have been reported in a series of cases using the TEP technique without mesh fixation [16,17]. In our series, a detailed analysis of our three patients with hernia recurrence showed that they were operated on in the first half of the study. There is no recurrence in patients in the second half of our study. This finding suggests that there is an important effect of increasing experience on hernia recurrence in video-endoscopic methods. In video-endoscopic surgery, the surgical field is limited, and the depth and sense of touch are decreased. Therefore, a specific learning curve is required to achieve successful results [18].

The European Hernia Association reported that the surgeon's learning curve for video-endoscopic hernia surgery is within a range of 50-100 cases [19]. In a series of 90 cases, the learning threshold was 30, whereas in a 700-case series this number was reported to be 60 [20,21]. Based on our observations, we can comment that there is a learning curve of 40-50 cases to reach the optimum level of knowledge of video-endoscopic anatomy, surgical dissection, and repair ability. In addition, the characteristics of hernia cases and related factors may be effective in the success of the video-endoscopic TEP method. Therefore, proper case selection should be considered during the initial learning process.

## Conclusions

Preoperative and early postoperative complications of video-endoscopic tension-free hernia repair via the TEP approach are extremely low. The recurrence rate is the most important indicator for successful hernia repair and is also acceptable. The success of TEP hernia repair increases in parallel with increases in the surgical experience. The results of our study with the TEP method are highly satisfactory and encouraging, especially when attention is paid to proper patient selection during the learning curve period.

## References

[1] Suguita FY, Essu FF, Oliveira LT (2017). Learning curve takes 65 repetition of totally extraperitoneal laparoscopy on inguinal hernias for reduction of operating time and complications. Surg Endosc.

[2] Tamme C, Scheidbach H, Hampe C, Schneider C, Köckerling F (2003). Totally extraperitoneal endoscopic inguinal hernia repair (TEP). Surg Endosc.

[3] Phillips EH, Carrol BJ, Fallas MJ (1993). Laparoscopic preperitoneal inguinal hernia repair without peritoneal incision. Surg Endosc.

[4] Xiaoqiang Z, Zhengni L, Jianfeng S, Tang R (2019). Triangle trocar configuration in laparoscopic totally extraperitoneal inguinal hernia repair: a prospective randomized controlled study. J Surg Res.

[5] Altın O, Kaya S (2018). Surgical results of total extraperitoneal hernia repair in 115 inguinal hernia patients. South Clin Ist Euras.

[6] Swadia ND (2011). Laparoscopic totally extraperitoneal inguinal hernia repair: 9 year's experience. Hernia.

[7] Meyer A, Blanc P, Balique JG (2013). Laparoscopic totally extraperitoneal inguinal hernia repair: twenty seven serious complications after 4565 consecutive operations. Rev Col Bras Cir.

[8] Messenger DE, Aroori S, Vipond MN (2010). Five-year prospective follow-up of 430 laparoscopic totally extraperitoneal inguinal hernia repairs in 275 patients. Ann R Coll Surg Engl.

[9] Morrison JE Jr, Jacobs VR (2008). Laparoscopic preperitoneal inguinal hernia repair using preformed polyester mesh without fixation: prospective study with 1-year follow-up results in a rural setting. Surg Laparosc Endosc Percutan Tech.

[10] Fitzgibbons RJ Jr, Camps J, Cornet DA (1995). Laparoscopic inguinal herniorrhaphy. Results of a multicenter trial. Ann Surg.

[11] Choi YY, Han SW, Bae SH, Kim SY, Hur KY, Kang GH (2012). Comparison of the outcomes between laparoscopic totally extraperitoneal repair and prolene hernia system for inguinal hernia; review of one surgeon's experience. J Korean Surg Soc.

[12] Pawanindra L, Kajla RK, Chander J, Saha R, Ramteke VK (2003). Randomized controlled study of laparoscopic total extra-peritoneal versus open lichtenstein inguinal hernia repair. Surg Endosc.

[13] Barrat C, Surlin V, Bordia A, Champault G (2003). Management of recurrent inguinal hernias: a prospective study of 163 cases. Hernia.

[14] Dulucq JL, Wintringer P, Mahajna A (2009). Laparoscopic totally extraperitoneal inguinal hernia repair: lessons learned from 3,100 hernia repairs over 15 years. Surg Endosc.

[15] Taylor C, Layani L, Liew V, Ghusn M, Crampton N, White S (2008). Laparoscopic inguinal hernia repair without mesh fixation: early results of a large randomised clinical trial. Surg Endosc.

[16] Garg P, Rajagopal M, Varghese V, Ismail M (2009). Laparoscopic total extraperitoneal inguinal hernia repair with nonfixation of the mesh for 1,692 hernias. Surg Endosc.

[17] Messaris E, Nicastri G, Dudrick SJ (2010). Total extraperitoneal laparoscopic inguinal hernia repair without mesh fixation. Arch Surg.

[18] Edwards CC 2nd, Bailey RW (2000). Laparoscopic hernia repair: the learning curve. Surg Laparosc Endosc Percutan Tech.

[19] Schouten N, Elshof JW, Simmermacher RK (2013). Selecting patients during the "learning curve" of endoscopic totally extraperitoneal (TEP) hernia repair. Hernia.

[20] Lim JW, Lee JY, Lee SE (2012). The learning curve for laparoscopic totally extraperitoneal herniorrhaphy by moving average. J Korean Surg Soc.

[21] Choi YY, Kim Z, Hur KY (2012). Learning curve for laparoscopic totally extraperitoneal repair of inguinal hernia. Can J Surg.

